# A Flexible Platform Containing Graphene Mesoporous Structure and Carbon Nanotube for Hydrogen Evolution

**DOI:** 10.1002/advs.201600208

**Published:** 2016-07-12

**Authors:** Rujing Zhang, Xiao Li, Li Zhang, Shuyuan Lin, Hongwei Zhu

**Affiliations:** ^1^ State Key Laboratory of New Ceramics and Fine ProcessingSchool of Materials Science and EngineeringTsinghua UniversityBeijing100084China; ^2^ Center for Nano and Micro MechanicsTsinghua UniversityBeijing100084China

**Keywords:** carbon nanotubes, graphene, hydrogen evolution, molybdenum sulfide

## Abstract

It is of great significance to design a platform with large surface area and high electrical conductivity for poorly conductive catalyst for hydrogen evolution reaction (HER), such as molybdenum sulfide (MoS*_x_*), a promising and cost‐effective nonprecious material. Here, the design and preparation of a free‐standing and tunable graphene mesoporous structure/single‐walled carbon nanotube (GMS/SWCNT) hybrid membrane is reported. Amorphous MoS*_x_* is electrodeposited on this platform through a wet chemical process under mild temperature. For MoS*_x_*@GMS/SWCNT hybrid electrode with a low catalyst loading of 32 μg cm^−2^, the onset potential is near 113 mV versus reversible hydrogen electrode (RHE) and a high current density of ≈71 mA cm^−2^ is achieved at 250 mV versus RHE. The excellent HER performance can be attributed to the large surface area for MoS*_x_* deposition, as well as the efficient electron transport and abundant active sites on the amorphous MoS*_x_* surface. This novel catalyst is found to outperform most previously reported MoS*_x_*‐based HER catalysts. Moreover, the flexibility of the electrode facilitates its stable catalytic performance even in extremely distorted states.

## 1 Introduction

With the rapid growth of world population and the ensuing intensified human activity, the development of sustainable and renewable energy source has become a vital issue in the modern world for maintaining a clean and livable environment. Hydrogen has high energy density and zero carbon emission, and is hence an ideal substitute of fossil fuels. Water splitting through electrochemical or photoelectrochemical processes is an attractive way to produce hydrogen. The best electrocatalytic material, Pt, can generate hydrogen at low overpotential.^1^ However, the scalable application of Pt is limited by its rare abundance and high production cost. Therefore, it is of great significance to develop highly efficient, stable, and cost‐effective electrocatalysts based on earth‐abundant materials.

Recent studies have identified molybdenum sulfides (MoS*_x_*) as promising catalysts for highly active hydrogen evolution reaction (HER) in acid solutions.^2^, ^3^, ^4^ For example, MoS_2_, a typical inexpensive alternative for Pt, has attracted a great deal of attention.^5^ The high activity mainly comes from the sulfur atoms at the edges of MoS_2_.^6^, ^7^ Extensive efforts have been dedicated to preparing edge‐rich MoS_2_ with ample electrochemically accessible active sites to enhance catalyst activity.^8^, ^9^, ^10^, ^11^, ^12^, ^13^, ^14^ Currently, the preparation of MoS_2_ for hydrogen evolution reaction involves complex and time‐consuming processes such as chemical Li‐intercalation and exfoliation,^15^, ^16^ chemical vapor deposition growth,^17^, ^18^ and hydrothermal/solvothermal reaction.^8^, ^19^, ^20^, ^21^, ^22^ In addition to MoS_2_, amorphous MoS*_x_* also has been proven to be efficient catalysts for hydrogenation and hydrodesulfurization.^23^, ^24^, ^25^ MoS*_x_* catalysts can be prepared through simple wet chemical processes, thus endowing them considerable advantage in terms of cost‐effective and scalable production.^24^

The electrochemical performance of MoS*_x_* catalysts can be improved not only by increasing active sites but also by employing suitable conductive substrates^26^ such as Au,^6^ carbon nanotubes (CNTs),^27^ carbon cloth,^13^, ^14^ ordered mesoporous carbon,^28^ and graphene.^8^, ^29^ Porous substrates have large surface area, which facilitates the nucleation of MoS*_x_* to give abundant active sites and contributes to their facile contact with the electrolyte. Highly conductive carbonaceous materials can also notably expedite electron transfer.^29^ Moreover, their stable chemical property in acid solution ensures stable catalysis. Graphene‐based structures prepared by using micelles as templates have shown high specific surface area and outstanding electrochemical performance.^30^, ^31^ However, the obtained precipitates are powders, which require complicated postprocessing to make electrodes. In addition, the electrical conductivity needs further improvement due to defects generated during the reduction of oxygen functional groups. Thus, exploring a suitable platform for MoS*_x_* catalysts with high specific surface area and excellent electrical conductivity remains a challenge.

Recently, flexible devices have attracted much attention due to their remarkable application in arbitrary shapes, such as flexible supercapacitors^32^ and touch screens.^33^ Such breakthroughs have inspired the design of novel electrodes for HER, i.e., to embed highly active catalysts on flexible electrodes that can work well in restricted space. To date, flexible electrodes for HER are rarely reported but highly desired.

In this work, we demonstrate a free‐standing and binder‐free hybrid membrane composed of graphene mesoporous structure (GMS) and single‐walled carbon nanotubes (SWCNTs) using Pluronic F127 micelles as soft templates. The porous membrane had large specific surface area and high electrical conductivity. By further anodic electrodeposition of amorphous MoS*_x_* on such platform, the achieved MoS*_x_*@GMS/SWCNT composite electrode exhibited impressive HER activity, with a current density reaching 71 mA cm^−2^ at an overpotential of 250 mV versus reversible hydrogen electrode (RHE). We show that the structure and composition of the hybrid membrane platform could be rationally controlled to regulate the resulting catalytic efficiency. Meanwhile, the good flexibility could allow its application in various distorted states.

## 2 Results and Discussion

**Figure 1:**
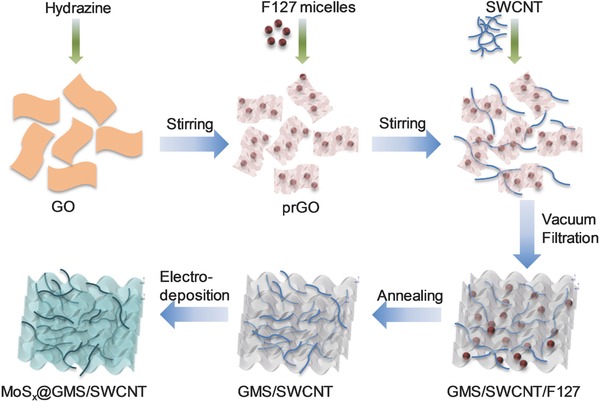
Schematic illustration of the synthetic method and structure of MoS*_x_*@GMS/SWCNT composite electrode.

The GMS/SWCNT hybrid platforms were prepared as illustrated in **Figure** **1**, and the detailed procedures are described in the Experimental Section. The main steps included the soft‐templating process and the introduction of SWCNTs. To begin with, F127 micelles were added into the suspension of partially reduced grapheme oxide (prGO) to serve as templates by associating the prGO nanosheets through hydrogen bonding.^30^ After complete reaction, SWCNT dispersion was added into the composite suspension. Then hybrid membranes were prepared through vacuum filtration, which was strong enough to be peeled from the filtration membrane directly. The F127 templates were completely removed during the subsequent annealing process in an inert atmosphere (Figure S1, Supporting Information), resulting in the hybrid membrane consisting of GMS and SWCNT. For comparison, GMS membrane and SWCNT membrane were also prepared.

**Figure 2:**
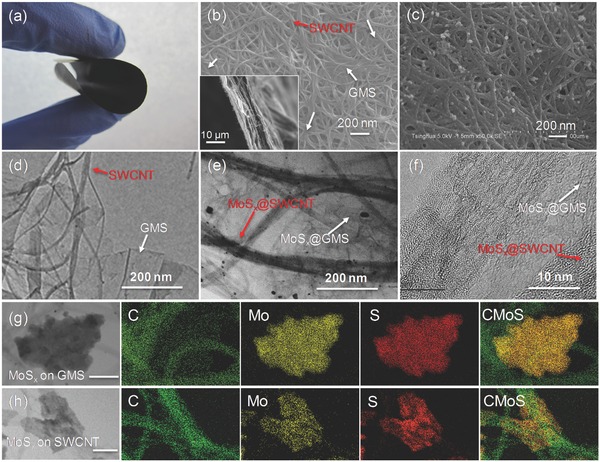
a) Digital photograph of a free‐standing and flexible GMS/SWCNT hybrid membrane. b) SEM image of GMS/SWCNT platform. The inset shows the cross section morphology. c) SEM image of MoS*_x_*@GMS/SWCNT composite electrode prepared with deposition time as 600 s. d) TEM image of GMS/SWCNT. e) TEM image of MoS*_x_*@GMS/SWCNT. f) HRTEM image of MoS*_x_*@GMS/SWCNT taken at 200 kV. EDX mapping of C, Mo, and S signal of MoS*_x_* on g) GMS and h) SWCNT. The scale bars in (g) and (h) are 100 nm. MoS*_x_*@GMS/SWCNT shown here was prepared with GO/SWCNT as 1:1 w/w and a catalyst deposition time of 600 s.

The GMS/SWCNT membrane possessed good mechanical strength and was uniformly assembled. It had good flexibility (**Figure** **2**a). Scanning electron spectroscopy (SEM) image shows the surface morphology of the hybrid platform, revealing the coexistence of GMS and SWCNTs in the membrane (Figure 2b). The thickness was ≈10 μm according to the cross‐section morphology shown in the inset. After electrochemical deposition, catalyst coating formed on the hybrid membrane and could be obviously observed (Figure 2c). The smooth surface of SWCNTs was coated with a rough catalyst layer, resulting in larger diameter (Figure S2, Supporting Information).

Figure 2d shows the typical transmission electron spectroscopy (TEM) image of GMS/SWCNT. SWCNTs were uniformly dispersed in small bundles without obvious aggregation. Large and thin graphene sheets derived from GMS were also observed. Figure 2e reveals the structure of MoS*_x_*@GMS/SWCNT, where the MoS*_x_* catalyst covered the surface of SWCNT bundles and GMS sheets. The high‐resolution TEM (HRTEM) image (Figure 2f) shows the amorphous form of MoS*_x_*. Relatively homogeneous distribution of MoS*_x_* on the scaffold was shown by energy dispersive X‐ray spectrometry (EDX) element mapping in Figure 2g,h, in the region of MoS*_x_* on GMS and SWCNT, respectively. The SEM, TEM, and EDX element mapping characterizations demonstrate the tight contact between the catalyst and the substrate, which could ensure effective charge transfer during the electrochemical process. Moreover, the interspace between SWCNTs facilitated the fast transport of reactants and products.

**Figure 3:**
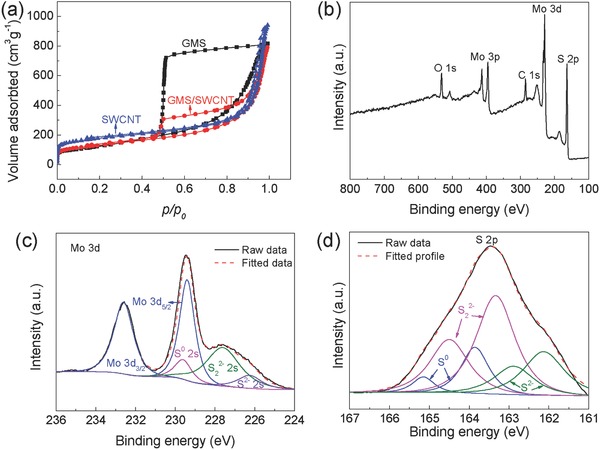
a) Nitrogen adsorption–desorption isotherms of the GMS, GMS/SWCNT hybrid membrane, and SWCNT. XPS spectra of MoS*_x_*@MGS/SWCNT: b) Survey spectrum, c) Mo3d, and d) S2p.

As an ideal platform for high‐efficient HER, it is important to provide large surface area for the exposure of active sites on catalyst. The microstructure of the GMS/SWCNT hybrid membrane platform was further explored by the nitrogen adsorption–desorption isotherm and the pore size distribution plot. The nitrogen adsorption–desorption isotherms of GMS membrane as well as the GMS/SWCNT hybrid membrane show typical IV isotherms, indicating the dominating presence of mesopores in the structure (**Figure** **3**a). The Brunauer–Emmett–Teller (BET) specific surface area of GMS and GMS/SWCNT membrane is 455.1 and 487.5 m^2^ g^−1^, respectively. And the pores mostly have a diameter of ≈4 nm (Figure S3, Supporting Information), which is in good accordance with previously reported mesoporous materials prepared with F127,^34^ demonstrating the effective templating process of F127 micelles. Based on the above discussion, we speculate that the introduction of SWCNTs not only enhanced the electrical conductivity of the hybrid membrane, but also prevented the agglomeration of prGO nanosheets and created a larger surface area.

In the typical two‐electrode electrodeposition system, [MoS_4_]^2−^ anions were attracted to the anode and the oxidation deposition proceeded as follows

(1) $${\left[{\mathrm{MoS}}_{4}\right]}^{2-}\to {\mathrm{MoS}}_{3}+\frac{1}{8}S_{8}+2e^{-}$$

The composition and bonding states of Mo and S were investigated by X‐ray photoelectron spectroscopy (XPS). As expected, the XPS survey spectrum shows C1s, O1s, Mo3d, and S2p peaks (Figure 3b). In the Mo3d spectrum, the main peaks are located at 232.6 and 229.4 eV, indicating the existence of Mo^6+^ ion in MoS_3_ (Figure 3c). The S2s peaks at 227.6 and 226.3 eV arise from the presence of disulfide and sulfide ions, respectively. Another peak at 229.6 eV suggests the existence of elemental sulfur. The S2p spectrum is fitted by three doublets, including elemental S^0^ at 163.9 eV, bridging S_2_^2−^ at 163.2 eV, and terminal S^2−^ located at 162.1 eV (Figure 3d). The atomic ratio of S to Mo is 3.4, corresponding to MoS_3_ plus elemental sulfur, in good accordance with Equation (1) and previous reports.^24^, ^29^

Figure S4a (Supporting Information) displays the results of Raman spectrum. Peaks at around 1347 and 1576 cm^−1^, as well as 2686 cm^−1^, are attributed to GMS and SWCNT. No obvious peaks of MoS*_x_* can be observed, indicating that the electrodeposited MoS*_x_* is amorphous. The absence of typical features of MoS*_x_* in the X‐ray diffraction (XRD) analysis also reveals the low crystallization of the catalyst (Figure S4b, Supporting Information), keeping consistent with the HRTEM result.

By tuning the structure of the hybrid platform or the catalyst loading on the surface, the catalytic activity can be easily controlled. Since the main process of the platform preparation includes the templating process of GMS and the introduction of SWCNT, one can manipulate the structure by changing the interaction between the template and the precursor, as well as by varying the content of SWCNT.

In the micelle‐templating process, the triblock copolymer micelle templates spontaneously connect with the prGO sheets by hydrogen bonding. Then, the micelle templates tended to be wrapped between the prGO layers, leading to efficient pore‐directing. However, pristine graphene oxide (GO) is strongly hydrophilic and this strong affinity with water can reduce its interaction with micelle templates.^30^ On the other hand, completely reduced GO sheets tend to reunite, limiting the effective insertion of micelles between the layers. In order to optimize the templating process, GO sheets were carefully reduced to obtain prGO with different oxygen content. The XPS spectra of prGO samples prepared with different reduction time under hydrazine treatment show significant decreases of oxygen‐containing functional groups (Figure S5, Supporting Information). The atomic oxygen content decreased from 32.6% to 13% after reaction for 2 h (Table S1, Supporting Information). After vacuum filtration, GMS membranes were obtained and used as substrates for MoS*_x_* deposition with a deposition time of 600 s. Figure S6 (Supporting Information) shows the polarization curves (*i*–*V* plots) of MoS*_x_*@GMS electrodes prepared with different reduction degrees. The optimal reduction time was found to be 45 min, corresponding to an oxygen content as 15.2%.

**Figure 4:**
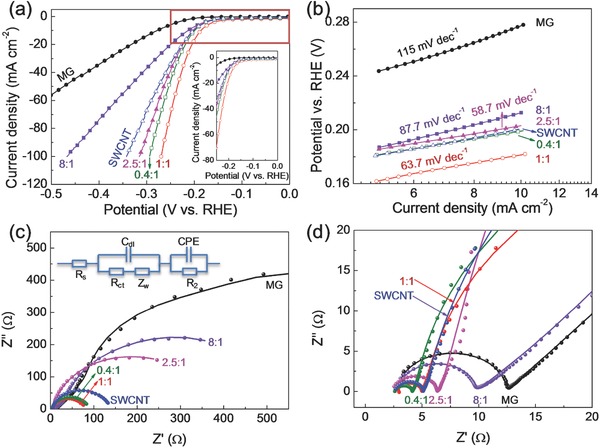
Electrochemical catalytic activities of MoS*_x_*@GMS/SWCNT with different SWCNT contents. The weight ratio of raw GO and SWCNT was 8:1, 2.5:1, 1:1, and 0.4:1. a) HER polarization curves. The inset shows the magnification of the red box in panel (a), with lower voltage window as ‐0.25–0 V. b) Corresponding Tafel plots. c,d) Nyquist plots of the hybrid electrodes with different SWCNT content. The inset in (c) shows the circuit model. (d) is the magnification of the high frequency region.

Using the optimal prGO dispersion which was reduced for 45 min, 10 μm thick membrane of pure GMS, pure SWCNT, and GMS/SWCNT composites (with initial GO/SWCNT ratios as 8:1, 2.5:1, 1:1, and 0.4:1 w/w) were investigated to determine the optimum composition of substrates for HER. Then, the MoS*_x_* catalyst was electrodeposited on the electrodes with a deposition time of 600 s. **Figure** **4**a presents the polarization curves of the samples with different GO/SWCNT weight ratios and the same catalyst loading. An optimal HER catalytic activity was obtained at a GO/SWCNT ratio of 1:1 w/w, which exhibited lower onset for HER and superior current density at an assigned voltage compared with other samples. The sample with the optimum SWCNT content was thus denoted as MoS*_x_*@GMS/SWCNTm (1:1). Its onset overpotential was 113.1 mV, much lower than that of other samples. Figure 4b reveals the Tafel slope of the optimal hybrid membrane catalyst as ≈63.7 mV dec^−1^, which is comparable to those of previously reported amorphous MoS*_x_* catalysts.^35^, ^36^ Electrochemical impedance spectroscopy (EIS) was carried out to investigate the electrode kinetics during catalysis process. From the Nyquist plots in Figure 4c,d, semicircles were observed in both the low‐ and high‐frequency range. A small series resistance (*R*_s_) was observed for all samples (≈2.8 Ω). The small resistance reveals low parasitic Ohmic losses, showing the importance of the direct deposition of catalyst on conductive substrates.^16^ Moreover, the plots demonstrate a decreased charge transfer resistance (*R*_ct_) with rising SWCNT content, implying faster HER kinetics. The fitted values of *R*_s_ and *R*_ct_ for all the samples were revealed in Table S2 (Supporting Information).

**Figure 5:**
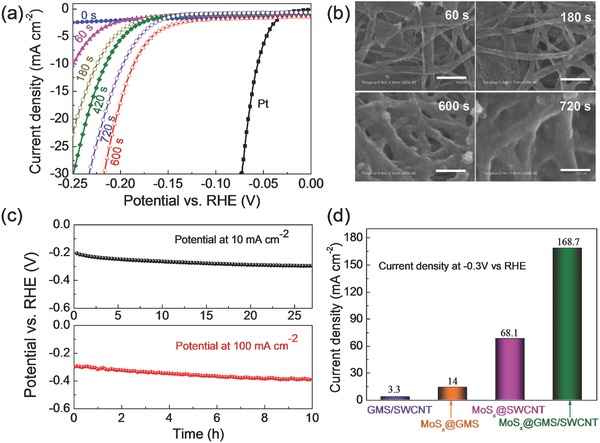
Electrochemical HER performance of MoS*_x_*@GMS/SWCNT prepared with different MoS*_x_* loadings. a) HER polarization curves and b) SEM images of MoS*_x_*@GMS/SWCNT with deposition time as 0, 60, 180, 420, 600, and 720 s. The scale labels in (b) are 100 nm. c) Electrochemical stability of the optimized MoS*_x_*@GMS/SWCNT composite electrode. The overpotential required to reach 10 mA cm^−2^ (upper) and 100 mA cm^−2^ (lower) remain low after long‐term reaction. d) Comparison of the electrocatalytic activity of GMS/SWCNT, MoS*_x_*@GMS, MoS*_x_*@SWCNT, and MoS*_x_*@GMS/SWCNT, revealing the synergistic effect of the three components in the hybrid catalyst.

Obviously, the catalytic activity of the hybrid catalyst depends on the loading of the electrochemically deposited amorphous MoS*_x_*. MoS*_x_*@GMS/SWCNT composite electrodes were prepared with optimized GO reduction time (45 min), optimal SWCNT content (1:1 w/w GO/SWCNT) and different catalyst loadings. Their polarization curves in 0.5 m H_2_SO_4_ aqueous solution at room temperature were measured (**Figure** **5**a). For comparison, the catalytic activity of Pt was also provided. The catalyst loading was controlled by tailoring the deposition time. The catalytic activity peaked when the deposition time was 600 s, corresponding to an optimal MoS*_x_* loading of 32 μg cm^−2^. SEM images in Figure 5b show how the morphology of MoS*_x_*@GMS/SWCNTm (1:1) varied with deposition time. Upon excessive loading, the pores become clogged by the catalyst, limiting the effective contact between the electrolyte and the catalyst. In addition, thicker amorphous MoS*_x_* restricts the efficient transfer of electron and proton,^35^ leading to the lower activity at high loading.

Long‐term stability is an important consideration for HER catalysts. As a commonly used protocol, long‐duration chronopotentiometric measurements were performed. After a long working period of 27 h, the overpotential required to drive 10 mA cm^−2^ current density increased only by ≈80 mV (Figure 5c, upper), which is comparable to previously reported results.^36^ Figure S7 (Supporting Information) shows the SEM images of MoS*_x_*@GMS/SWCNT composite electrode before electrocatalysis and after working for 27 h, with the coating remaining almost the same on the scaffold. Also, no obvious catalyst degradation was observed at a high constant current density of 100 mA cm^−2^ (Figure 5c, lower). Figure S8 (Supporting Information) shows the electrochemical stability of the composite electrode at a constant potential of 221 mV versus RHE. Although the stability of this amorphous catalyst is not as excellent as some other MoS*_x_* materials with good crystallization, the catalytic activity after cycling is reasonable and acceptable compared with previously reported analogues. In addition, the overpotential required to attain 100 mA cm^−2^ remained low. Figure 5d shows that the current densities of MoS*_x_*@GMS/SWCNT, MoS*_x_*@SWCNT, MoS*_x_*@GMS, and GMS/SWCNT at 0.3 V versus RHE are 168.7, 68.1, 14, and 3.3 mA cm^−2^, respectively. This comparison indicates the synergy of the three components in MoS*_x_*@GMS/SWCNT composite electrode, providing a significant enhancement of the electrocatalytic performance. The nanostructure is efficient for enhancing electrocatalyst performance, in good accordance with the strategy prediscussed by Faber et al.^37^

**Figure 6:**
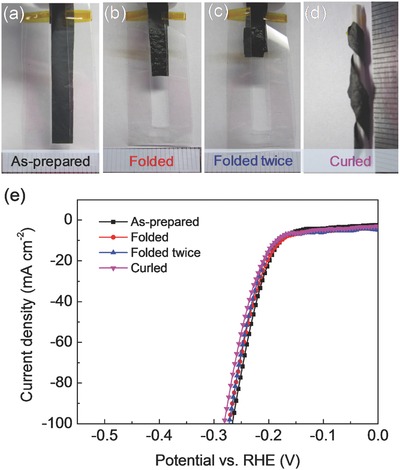
a–d) MoS*_x_* @GMS/SWCNT at different deformation states, including straight hanging, twofolds, threefolds, and spiral state. e) Polarization curves of MoS*_x_*@GMS/SWCNT at different deformation states.

The good flexibility of the MoS*_x_*@GMS/SWCNT composite membrane enables its diverse deformations without obvious structural damage (**Figure** **6**a–d). Figure 6e shows the polarization curves of the hybrid membrane under different distorted states as shown in Figure 6a–d, demonstrating relatively stable electrocatalytic performance. The steady catalytic activity facilitates its potential applications in limited space, complex reaction unit, and extreme environment.

Additionally, the HER performance of our MoS*_x_*@GMS/SWCNT was compared with some reported nonprecious HER catalysts (**Table** **1**). In general, compared with other systems, our catalyst could produce higher current density, obviously lower onset potential, and notably low catalyst loading. As discussed above, the high HER efficiency of MoS*_x_*@GMS/SWCNT can be attributed to the following reasons: (1) The porous GMS/SWCNT platform provides large surface area for the deposition of MoS*_x_* to sufficiently expose active sites. The numerous pores in the substrate also facilitate the easy diffusion of electrolyte and fast transport of reactants and products. (2) The platform has good electrical conductivity and maintains intimate contact with MoS*_x_*, which ensures the effective transport of electrons during the electrochemical process. (3) Plenty of uncoordinated sulfur atoms exist over the entire surface of amorphous MoS*_x_* to serve as the active sites for the HER.^24^, ^29^ For example, XPS characterization has confirmed the existence of bridging S_2_^2−^ and apical S^2−^ on the surface of the amorphous catalyst. (4) The porous structure is beneficial for the electrocatalytic performance through expediting the timely release of small H_2_ bubbles formed during the HER process (Movie S1, Supporting Information), similar to the previously reported nanostructured MoS_2_ catalyst.^38^

**Table 1:** Comparison of HER performance of MoS*_x_*@GMS/SWCNT composite electrode with other reported nonmetal electrocatalysts in acidic aqueous electrolyte

Catalyst	Loading amount [mg cm^−2^]	Onset overpotential [mV] versus RHE	Overpotential at 20 mA cm^−2^ [mV] versus RHE	Current density at −250 mV versus RHE [mA cm^−2^]	Ref.
MoS*_x_*@GMS/SWCNT	0.032	113	204	71	This work
MoS_2_/RGO hybrid catalyst on carbon fiber paper	1	≈100	≈180	–	8
Pristine MWMoS_2_@MWCNT	0.05	150–200	–	≈1	39
Li electrochemically intercalated MoS_2_ on carbon fiber paper	0.12	≈113	≈180	–	10
Amorphous molybdenum sulfide on dealloyed nanoporous gold	0.006	≈125	≈230	≈25	40
MoS*_x_* on 3D graphene‐protected Ni foam	8.09	109–141	≈160	≈87	29
MoS_2_ on carbon cloth	0.19	≈100	≈200	86	14
WS_2_ on carbon cloth	1.5	≈150	≈280	15	
MoS_2_ nanoflower on rGO paper	0.116	190	≈350	≈10	19
MoS_2_ nanoparticles on carbon nanofiber foam	–	120	≈240	≈25	20
Nanostructured MoS_2_/Ti	0.12	150	≈240	≈30	38
WS_2_/rGO	0.40	150–200	≈290	≈5	41
MoS*_x_*Cl*_y_* grown on vertical graphene; measured on a rotating disk electrode	–	≈125	≈175	–	42
48 h oven exfoliated WS_2_ on graphite disk	1.0 ± 0.2	75	≈170	–	43
Metallic‐phase MoS_2_	0.043	≈130	≈225	≈35	44

## 3 Conclusion

In summary, a free‐standing and binder‐free hybrid membrane composed of GMS and SWCNTs was prepared through templating with Pluronic F127 micelles. The composite membrane possessed high specific surface area and remarkable electrical conductivity. Electrodeposition of amorphous MoS*_x_* on this composite platform yielded a novel catalyst with outstanding HER catalytic activity. The MoS*_x_*@GMS/SWCNT composite electrode for HER could be tuned by adjusting the interaction of templates with prGO, the content of SWCNTs, as well as the loading amount of MoS*_x_*. Moreover, the good flexibility and steady HER performance in extremely distorted states could enable the electrodes for use in many fields. This material is a simple, cost‐effective, and scalable nonprecious HER catalyst because it can be prepared from an easy wet chemical process at a mild temperature with low MoS*_x_* loading. We also believe the GMS/SWCNT hybrid architecture can serve as an excellent platform for other catalytic processes and pave a new way for designing novel electrodes for other gas evolution reactions.

## 4 Experimental Section

*Materials and Chemicals*: Natural graphite was purchased from Qingdao Henglide Graphite Co., Ltd. GO was prepared by a modified Hummers method.^45^ SWCNTs were purchased from Nanjing XFNANO Materials Tech Co., Ltd. Triblock copolymer Pluronic F127, EO_106_PO_70_EO_106_ (EO, ethylene; PO, propylene) and ammonium tetrathiomolybdate ((NH_4_)_2_MoS_4_, 99.97%) were purchased from Sigma‐Aldrich and used as received.

*Preparation of GMS/SWCNT Hybrid Membrane*: First, 7.5 mL GO aqueous suspension (2 mg mL^−1^) was mixed with 15 μL hydrazine at 90 °C for 45 min under vigorous stirring. Then 3 mL F127 solution (10 wt%) was added into the partially reduced GO solution. After stirring for 2 h, 2 mL HCl (37 wt%) was added with stirring for another 2 h to form GMS dispersion. Then 15 mL SWCNT aqueous dispersion (1 mg mL^−1^) was added to the GMS solution. The liquid system was stirred vigorously for 12 h. Then it was divided equally into two parts for subsequent vacuum filtration process. The obtained GMS/SWCNT/F127 hybrid membrane was dried at 80 °C and then transferred into a furnace for annealing at 350 °C for 2 h and 900 °C for 1 h in Ar atmosphere to achieve GMS/SWCNT membrane.

The reduction degree of GO was tuned by changing the reduction time, such as 0, 15, 30, 45, 60, and 120 min. In addition, the hybrid structure was regulated by controlling the volume of SWCNT dispersion. The weight ratio of GO:SWCNT was adjusted as 8:1, 2.5:1, 1:1, and 0.4:1. In this work, all membranes used were controlled with a thickness of ≈10 μm. Pure GMS and SWCNT membranes were also prepared for comparisons.

*Electrodeposition of Amorphous Molybdenum Sulfides*: The electrodeposition process was carried out using a typical two‐electrode system. A piece of GMS/SWCNT membrane (20 mm × 5 mm) was used as the anode while a platinum foil was used as the cathode. The system worked in a 1 × 10^−3^ m (NH_4_)_2_MoS_4_ solution (solvent: *N*,*N*‐dimethylformamide). Amorphous MoS*_x_* was electrodeposited on the hybrid membrane using a constant anodic current density of 0.2 mA cm^−2^. Finally, the electrode was rinsed with distilled water and dried at 80 °C. The loading was controlled by tuning the deposition time as 60, 180, 420, 600, 720 s.

*Characterizations*: Morphology characterization was performed using SEM (FEI QUANTA 450 FEG) and TEM (JEM 2010). Chemical characterization was carried out with thermal gravity analysis (NETZSCH STA 449F3), XRD (Bruker AXS D8 ADVANCE), and XPS (ESCALAB 250Xi). Nitrogen adsorption–desorption isotherm and pores distribution were measured with an Aurosorb‐iQ2‐MP analyzer using BET method.

*Electrochemical Measurements*: All electrochemical experiments were conducted in a three‐electrode system using a CHI 660E electrochemical workstation. The electrolyte was 0.5 m H_2_SO_4_ with a Ag/AgCl electrode as the reference electrode, a Pt foil as the counter electrode. The flexible and mechanically robust MoS*_x_*@GMS/SWCNT hybrid membrane was used as the working electrode directly, with an effective working area of 1 cm × 0.5 cm. Linear sweep voltammetry curves were obtained with a scan rate of 5 mV s^−1^. EIS was conducted at the overpotential of −150 mV with frequencies ranging from 1 MHz to 0.01 Hz. All the polarization curves were *iR* corrected. The EIS spectra were fitted by the Z‐view software.

## Supporting information

As a service to our authors and readers, this journal provides supporting information supplied by the authors. Such materials are peer reviewed and may be re‐organized for online delivery, but are not copy‐edited or typeset. Technical support issues arising from supporting information (other than missing files) should be addressed to the authors.

Supplementary

Supplementary
