# Supplementary material for: A Flexible Platform Containing Graphene Mesoporous Structure and Carbon Nanotube for Hydrogen Evolution
Source: Adv Sci (Weinh). 2016 Jul 12;3(11):1600208. doi: 10.1002/advs.201600208 (PMC5102666; doi:10.1002/advs.201600208)
Supplement: Supplementary file 1 — Supplementary [file ADVS-3-0o-s001.pdf]

Copyright WILEY-VCH Verlag GmbH & Co. KGaA, 69469 Weinheim, Germany, 2016.

ADVANCED SCIENCE
Open Access

Supporting Information

for *Adv. Sci.,* DOI: 10.1002/advs.201600208

A Flexible Platform Containing Graphene Mesoporous Structure and Carbon Nanotube for Hydrogen Evolution

*Rujing Zhang, Xiao Li, Li Zhang, Shuyuan Lin, and Hongwei Zhu**

Copyright WILEY-VCH Verlag GmbH & Co. KGaA, 69469 Weinheim, Germany, 2016.

Supporting Information

A Flexible Platform Containing Graphene Mesoporous Structure and Carbon Nanotube for Hydrogen Evolution

*Rujing Zhang, Xiao Li, Li Zhang, Shuyuan Lin, and Hongwei Zhu**

Index

Figure S1. TGA curves of F127 and freshly obtained GMS/SWCNT/F127 membrane by vacuum filtration.

Figure S2. High resolution SEM images of membrane before and after electrodeposition

Figure S3. Pore size distribution of platforms.

Figure S4. Raman spectra and XRD patterns of membranes.

Figure S5. XPS spectra of prGO with different reduction degree.

Figure S6. Polarization curves of MoS_x@GMS prepared with different reduction degree.

Figure S7. SEM images of composite electrode before and after electro-catalysis.

Figure S8. Electrochemical stability of MoS_x@GMS/SWCNT composite electrode at a constant potential of 221 mV *vs*. RHE.

Table S1. Oxygen content of prGO prepared with different reduction time.

Table S2. EIS parameters of MoS_x@GMS/SWCNT composite prepared with different SWCNT content.

Movie S1. HER process of MoS_x@GMS/SWCNT in 0.5M H_2SO4.

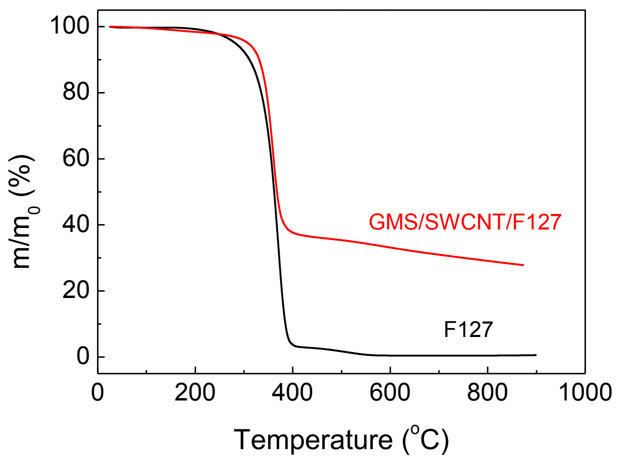

Figure S1. TGA curve of F127 (black line)and freshly obtained GMS/SWCNT/F127 hybrid membrane by vacuum filtration (red line), showing that F127 was completely removed during the annealing process.

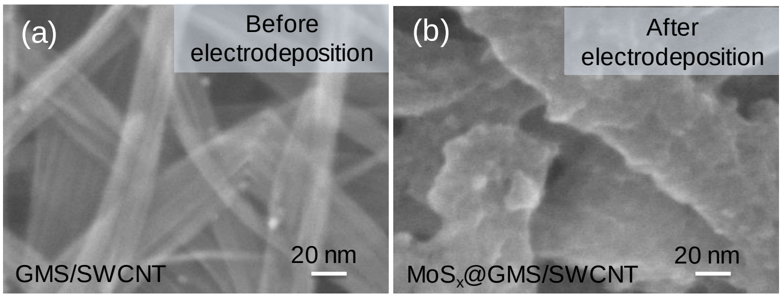

Figure S2. High resolution SEM images of (a) GMS/SWCNT platform and (b) MoS_x@GMS/SWCNT composite electrode.

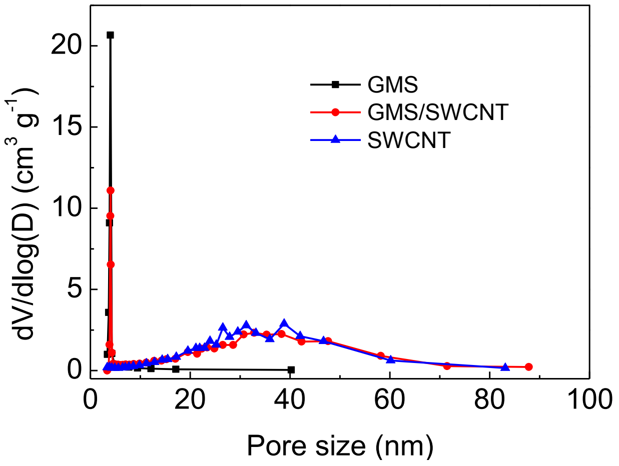

Figure S3. Pore size distributions of GMS membrane, GMS/SWCNT hybrid platform and SWCNT membrane.

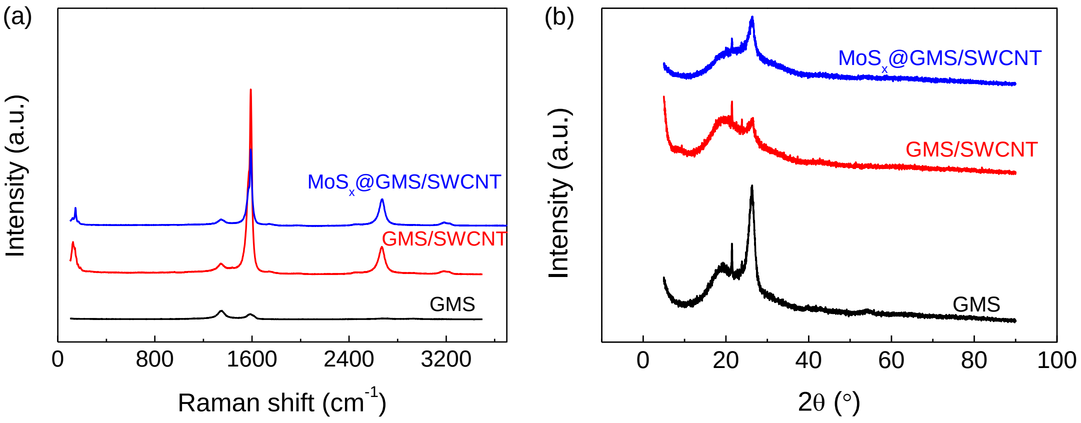

Figure S4. (a) Raman spectra and (b) XRD patterns (b) of the synthesized GMS, GMS/SWCNT platform and MoS$_x$@GMS/SWCNT.

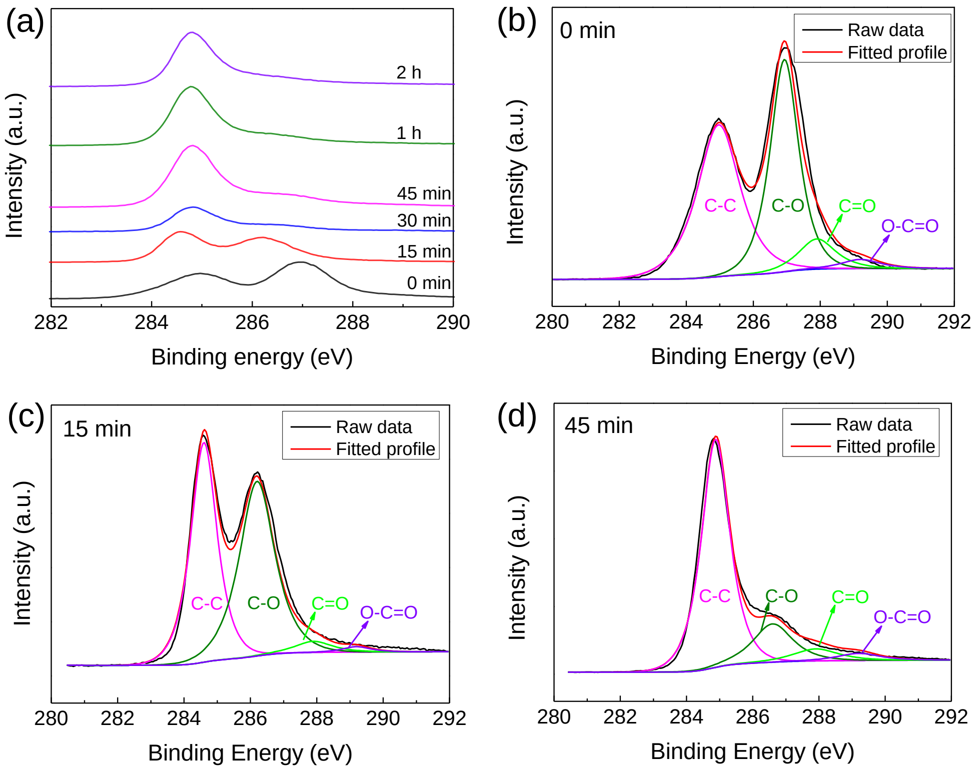

Figure S5. XPS spectra of prGO prepared with different reduction time. (a) Comparison of XPS spectra. (b) 0 min. (c) 15 min. (d) 45 min.

Table S1. Oxygen content of prGO prepared with different reduction times.

Reduction time (min)	0	15	30	45	60	120
C/O atomic ratio	2.07	2.92	4.92	5.6	6.34	6.67
Oxygen content (at.%)	32.6	25	16.9	15.2	13.6	13

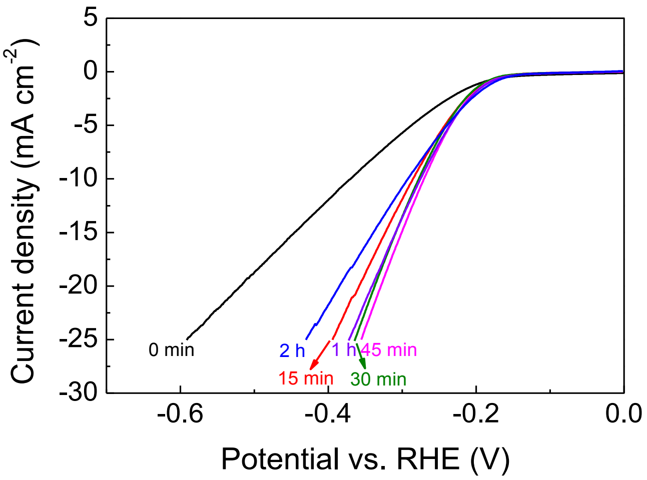

Figure S6. Polarization curves of MoS_x@GMS prepared with different reduction degree. In the preparation process of GMS, prGO nanosheets with different reduction time were used, *i.e.* 0, 15, 30, 45, 60 and 120 min.

Table S2. EIS parameters of MoS_x@GMS/SWCNT composite prepared with different SWCNT content.

Samples	R_s (Ω)	R_{ct} (Ω)
GMS	2.885	9.492
8:1	2.891	6.814
2.5:1	2.507	3.740
1:1	2.872	2.361
0.4:1	2.682	1.417
SWCNT	2.687	1.901

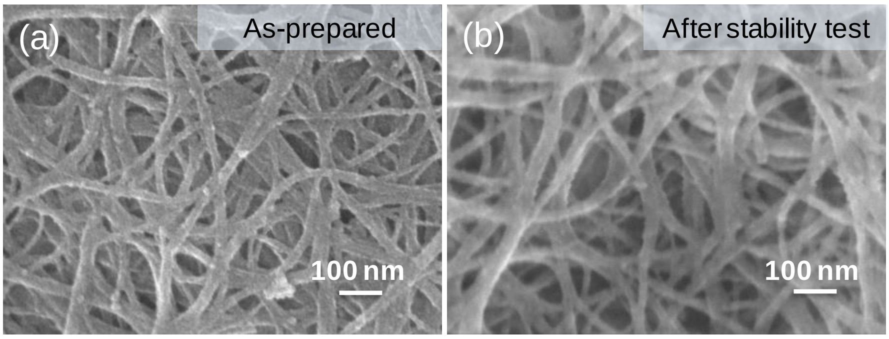

Figure S7. SEM images of MoS_x@GMS/SWCNT composite electrode (a) before catalysis process and (b) after stability test for 27 h at current density of 10 mA cm^{-2}.

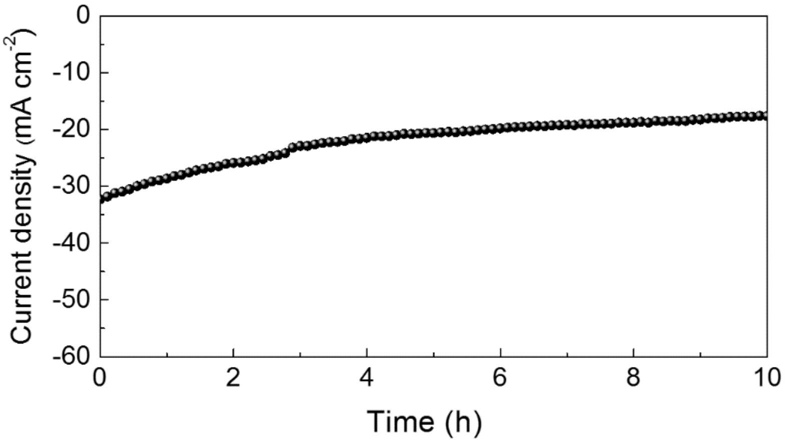

Figure S8. Electrochemical stability of MoS_x@GMS/SWCNT composite electrode at a constant potential of 221 mV *vs*. RHE.
